# Fluid intake of Latin American adults: results of four 2016 Liq.In^7^ national cross-sectional surveys

**DOI:** 10.1007/s00394-018-1724-z

**Published:** 2018-06-01

**Authors:** H. Martinez, C. Morin, J. Gandy, E. Carmuega, J. L. Arredondo, C. Pimentel, L. A. Moreno, S. A. Kavouras, J. Salas-Salvadó, I. Guelinckx

**Affiliations:** 1 Hospital Infantil de México Federico Gómez, Mexico City, Mexico; 2 Department of Hydration and Health, Danone Research, Route Départemental 128, 91767 Palaiseau, France; 3 British Dietetic Association, Birmingham, UK; 4 School of Life and Medical Sciences, University of Hertfordshire, Hatfield, UK; 5 Center of Studies on Infant Nutrition (CESNI) Buenos Aires, Buenos Aires, Argentina; 6 Unidad de Apoyo a la Investigación Clínica, Instituto Nacional de Pediatría, Mexico City, Mexico; 7 GENUD (Growth, Exercise, NUtrition and Development) Research Group, Faculty of Health Sciences, Universidad de Zaragoza, Instituto Agroalimentario de Aragón (IA2), Instituto Investigación Sanitaria Aragón (IIS Aragón) Zaragoza, Zaragoza, Spain; 8 CIBERobn (Centro de Investigación Biomédica en Red Fisiopatología de la Obesidad y Nutrición), Institute of Health Carlos III, Madrid, Spain; 9 Hydration Science Lab, University of Arkansas, Fayetteville, AR USA; 10 Division of Endocrinology, University of Arkansas for Medical Sciences, Little Rock, AR USA; 11 Human Nutrition Unit, Biochemistry and Biotechnology Department, Faculty of Medicine and Health Sciences, Hospital Universitari de Sant Joan de Reus, IISPV (Institut d’Investigació Sanitària Pere Virgili), Universitat Rovira i Virgili, Reus, Spain

**Keywords:** Beverages, Fluid intake, Hydration, Liq.In^7^, Water, Argentina, Brazil, Mexico, Uruguay

## Abstract

**Purpose:**

To report total fluid intake (TFI) and the intake of different fluid types in adults (≥ 18 years old) from Mexico, Argentina, Brazil and Uruguay. To compare intakes between countries and with recommended adequate intake (AI) of water from fluids.

**Methods:**

Cross-sectional data were collected using a validated liquid intake 7-day record (*Liq.In*^*7*^) in populations from Argentina (*n* = 1089), Brazil (*n* = 477), Mexico (*n* = 1677) and Uruguay (*n* = 554). Population characteristics, including age, gender, body mass index and socioeconomic level were recorded. Mean TFI was compared with the AI of water from fluids set by the USA Institute of Medicine.

**Results:**

The lowest TFI was recorded in Mexican women (1748 mL/day) and the highest in Argentinean men (2318 mL/day). Median daily TFI was significantly different between countries; Uruguay and Argentina had higher values than Mexico and Brazil. In the former, plain water contributed to only 25% of TFI, the remainder being predominantly from hot beverages. Approximately, a third of adults did not drink enough fluid to meet the recommended AI. High SSB consumption was reported, which was significantly different between countries (*p* < 0.05), the highest being in Mexico (median 25–75th percentiles): 531 (300–895 mL/day.

**Conclusions:**

This survey highlights the need to increase water consumption and reduce SSB intake in this region to avoid potential associated health risks. These findings may be useful information in monitoring public health policy strategies.

**Electronic supplementary material:**

The online version of this article (10.1007/s00394-018-1724-z) contains supplementary material, which is available to authorized users.

## Introduction

The role of water consumption and optimal body hydration is becoming a subject of great scientific interest. While the effects of severe dehydration are well documented, [1] it is only recently that the effects of low water consumption are being investigated. Regular low (< 1.2 L/day) drinkers are able to maintain normal plasma osmolality by reducing body water losses via urine; this results in low, highly concentrated urinary output [2]. Low drinkers may have increased levels of copeptin, a precursor of vasopressin, the anti-diuretic hormone responsible for fluid homeostasis and urine concentration [3]. Increased levels of copeptin have been linked to cardiometabolic diseases such as type 2 diabetes and heart disease [4, 5]. In addition, increasing water intake has been shown to attenuate copeptin levels [6].

It is therefore a concern that a significant proportion of adults in Latin American countries have been shown to consume less than the recommended intake of water from fluids [7]. In a survey of 13 countries worldwide, 57% of Mexican adults did not meet the recommended intake levels; 41% of Brazilian adults and 36% of Argentinian adults were also shown to consume less than the recommendations [7]. In this former analysis, the European Food Safety Authority (EFSA) reference values for total water intake [8], adjusted to account for water from food, were used, as countries from all around the world were included. In addition, the EFSA recommendations are more conservative than the reference values set by the USA Institute of Medicine (IOM) [9] and therefore unlikely to overestimate the number of people not adhering to recommendations. Country-specific recommendations for water intake are not available in Latin American countries. Therefore, for future comparisons it would be more appropriate to use the adequate intake of water set by the USA IOM [9], in line with other Latin American studies [10]. It is now apposite to resurvey fluid consumption in Latin American countries and, as only countries from this region were surveyed, to use the IOM recommendations [9] for comparison purposes.

In addition to the links between low fluid consumption and chronic diseases, the type of fluids consumed is also important. In particular, the consumption of sugar-sweetened beverages (SSB) has been shown to be linked to obesity [11], type 2 diabetes [12] and cardiovascular disease [13]. Consumption of SSB in Latin America is among the highest in the world [14]. Given the increasing levels of obesity in Latin America, this raises additional cause for concern [15]. The average body mass index (BMI) of Latin Americans increased twice as fast as the global average between 1980 and 2008, with 70% of Mexicans now considered either overweight or obese [16]. Unsurprisingly, this has been accompanied by a rapid increase in associated conditions such as type 2 diabetes, metabolic syndrome and cardiovascular disease [17–19]. It is vital that policy makers understand the drivers behind this epidemic and how best to intervene to change behaviors in view of the societal and financial costs of such widespread health problems.

There is a lack of information in relation to the amount and type of fluids consumed around the world and specifically in Latin America. Therefore, the primary aim of the present study was to report total fluid intake (TFI) and intake of different fluid types of adults (≥ 18 years) in Argentina, Brazil, Mexico and Uruguay, using a validated 7-day fluid record (the *Liq.In*^*7*^ diary) [20]. The secondary aims were to make between-country comparisons and association with the IOM recommendations on adequate intake (AI) of water from fluids [9].

## Methods

### Design and study population

**Fig. 1:**
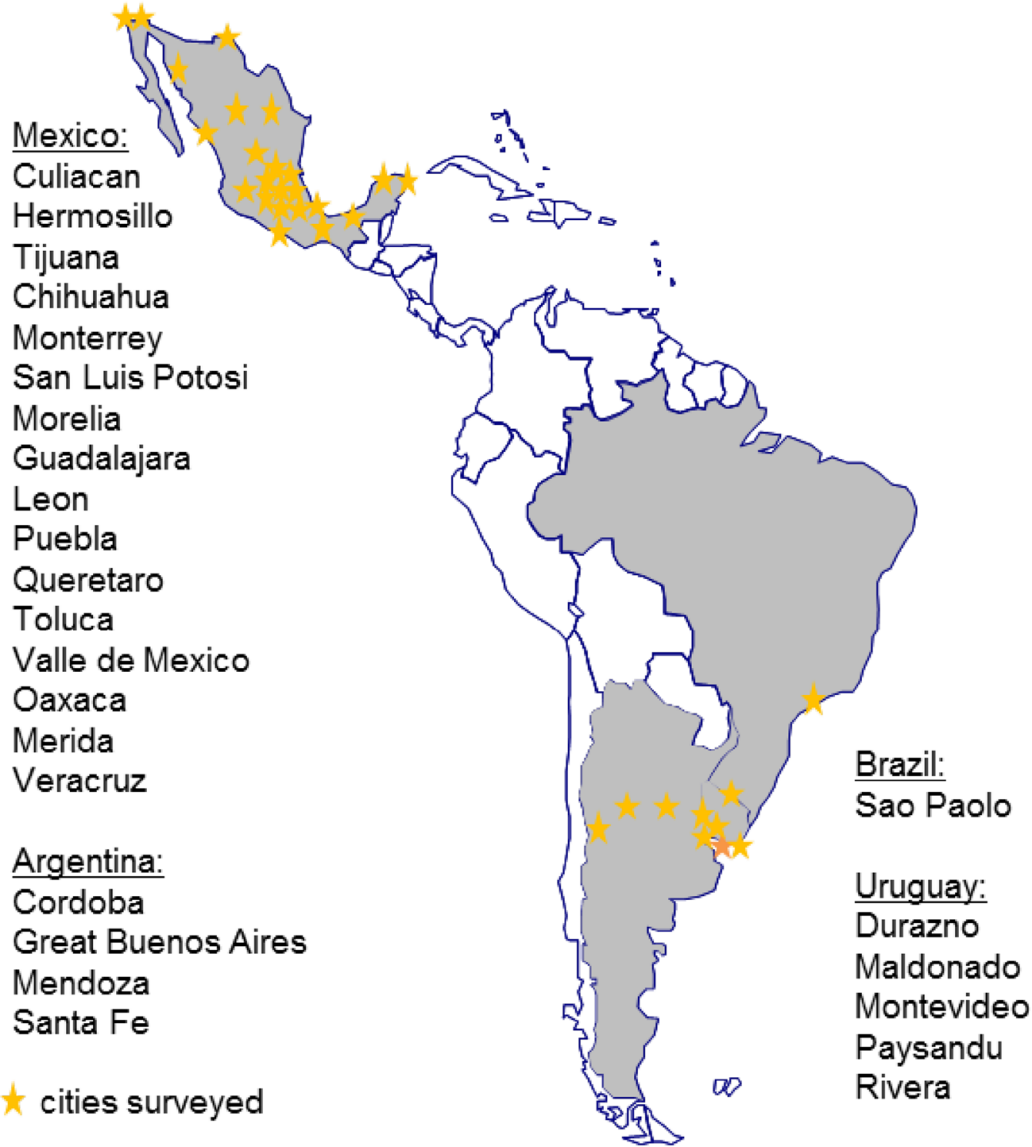
Map showing the cities in each country from where participants were recruited

The present analysis reports cross-sectional surveys of adults (≥ 18 years) in four Latin America countries: Argentina, Brazil, Mexico and Uruguay. The method of recruitment, the instruments for data collection and data treatment were harmonized across the surveys. The data collection was performed between March and May 2016 in urban areas in different regions of Argentina, Mexico and Uruguay. For Brazil, data were only collected in the City of São Paulo between November and December 2016. Figure 1 shows participants’ city of residence. Recruitment of participants in each country was performed via a door-to-door recruitment until suitable quotas for age, gender, region and socioeconomic characteristics, in relation to the total country population, were met. To determine the socioeconomic status (SES) of participants, the Asociación Mexicana de Agencias de Investigación de Mercado y Opinión Pública (AMAI) system was used in Mexico, Argentina and Uruguay, and the ABEP classification in Brazil [21, 22]. Both systems use a combination of the following criteria to determine SES: work status, occupation, education, medical coverage, number of domestic servants, number of bathrooms, household equipment, ownership of an international credit card and/or access to public services (e.g., water, type of street). For data analysis, SES classes were harmonized as detailed in supplementary table S1.

Only one individual per household was eligible to participate. Apparently healthy individuals were eligible, except individuals working in any capacity in a company associated with the manufacture, distribution and/or sale of water and any other kind of beverage were excluded from participation. Pregnancy and/or lactation were not exclusion criteria.

### Ethical considerations

The survey protocol was reviewed and approved by the University of Arkansas Review Board (ref. 14-12-376). After receiving a detailed description of the study and its objectives, following the principles of informed consent, participants gave oral approval of their willingness to be included. No monetary incentive was offered for taking part in the study. All data were recorded anonymously.

### Anthropometry

Height (m) and weight (kg) were self-reported by participants; BMI was calculated as kg/m^2^ [23].

### Assessment of total fluid intake and its composition

Participants were provided with the *Liq.In*^*7*^ record, a 7-day fluid-specific record validated for accuracy and reliability [20] in the official language of the country. Before the survey began, the researcher explained how to complete the record in an initial face-to-face interview in the participant’s home. After a period of 7 days, the record was collected by the researcher and checked for completion with the participant. The *Liq.In*^*7*^ is structured around times of the day from waking up, meal times (breakfast, lunch, dinner), periods between meals (morning, before lunch/aperitif, afternoon, tea break, before dinner/aperitif, just before going to bed) and during the night. The participants were instructed to report all drinking events at any moment of the day with the following details: the fluid type, the volume consumed, the size of the container from which it was drunk, where it was consumed and whether food was also consumed. However, the type of food consumed was not reported. To assist estimation of the amount of fluid consumed, a booklet with pictures of standard fluid containers was provided.

### Classification and analysis of the fluid types

Fluids were classified as water (tap and bottled water), milk and milk derivatives, hot beverages (coffee, tea and other), 100% fruit juices, sugar-sweetened beverages (SSB) (carbonated soft drinks (CSD), juice-based drinks, functional beverages such as energy and sports drinks, ready to drink tea and coffee and flavored water), artificial/non-nutritive sweeteners beverages (A/NSB) (diet/zero/light soft drinks), alcoholic beverages and other beverages. More details are given in supplementary table S2. Total fluid intake was defined as the sum of all these categories. In Uruguay and Argentina, the *Liq.In*^*7*^ record had a specific code for Maté (a hot infusion of *Ilex paraguayensis*, which is a cultural beverage largely consumed in Southeast Latin America), as previous surveys had indicated that this beverage heavily influenced median daily TFI. An individual was defined as a consumer of a certain fluid type if this fluid type was consumed at least once during the 7-day period. Individual’s daily TFI was compared with the adequate intake of water from fluids set by the USA IOM [9]. To allow comparison with previously published data, the comparison between observed intakes and the recommendations set by EFSA [8] is also provided in supplementary figure S2.

The proportion of individuals drinking ≤ 1 serving (250 mL) of SSB per week, 2–6 servings of SSB per week and ≥ 1 serving/day intake of SSB per day was calculated. These cutoffs were obtained from meta-analyses associating such levels of intake with potential risks for the development of obesity, type 2 diabetes and metabolic syndrome [12, 24, 25].

### Statistical analysis

The demographic and anthropometric characteristics of the study population are presented either as means and standard deviation for continuous variables, or numbers and percentages for dichotomous variables. Due to the skewed distribution in intake data, as shown in supplementary Figure S1, TFI is presented as median with percentiles as well as mean and standard error of mean. Different fluid types are recorded as median (50th percentile) [25th–75th percentiles (P25-P75)] and proportion of consumers. The mean [standard error of mean (SEM)] of the different fluid types can be found in supplementary table S4a–d. The contribution of each fluid type to TFI was calculated using the mean values for TFI. Between groups, comparisons were made with a Wilcoxon rank test for continuous variables. A Bonferroni post hoc test was used to correct for multiple comparisons. All statistical tests were two-tailed and the significance level was set at *p* < 0.05. All analyses were performed using the SPSS software version 22.0 (SPSS Inc., Chicago, IL) and were verified by a statistician.

## Results

The TFI and type of fluids consumed were analyzed for nearly 4000 adults from Argentina (*n* = 1089), Brazil (*n* = 477), Mexico (*n* = 1677) and Uruguay (*n* = 554). The general characteristics of the study population, by country, are shown in Table 1. There was a slight predominance of female participants (55%). The mean age in each of these countries was below 40 years and the number of respondents in each age group was similar. The percentage of overweight and obese participants was higher in Brazil, Argentina and Mexico than in Uruguay; in Brazil and Mexico approximately 35% of the sample population were of normal weight compared with more than 65% in Uruguay. With respect to socioeconomic classification, the Brazilian participants were all from SESs AB and C. In Argentina and Mexico, approximately 60% of the participants were from SESs AB and C; 48% of Uruguayans were from these SESs.

**Table 1:** Demographic and anthropometric characteristics of the study population, by country

Country	Mexico (*n* = 1677)		Brazil (*n* = 477)		Argentina (*n* = 1089)		Uruguay (*n* = 554)	
Sample size (*N*, %)								
Men	746	(44)	224	(47)	464	(43)	278	(50)
Women	931	(56)	253	(53)	625	(57)	276	(50)
Age (years)^a^	37.6	± 13.1	39.5	± 14.0	38.5	± 13.3	37.7	± 13.3
Age group								
18–25 years	397	(24)	98	(21)	234	(21)	135	(24)
26–35 years	411	(25)	108	(23)	277	(25)	124	(22)
36–50 years	553	(33)	142	(30)	329	(30)	167	(30)
> 50 years	316	(19)	129	(27)	249	(23)	128	(23)
Weight (kg)^a^	71.4	± 14.0	75.9	± 16.2	72.7	± 15.8	73.3	± 22.7
Height (m)^a^	1.6	± 0.1	1.7	± 0.1	1.7	± 0.1	1.7	± 0.1
BMI (kg/m^2^)^a^	26.7	± 4.9	27.0	± 6.8	26.0	± 5.6	25.7	± 10.3
Socioeconomic level (*N*, %)								
AB	125	(7)	210	(56)	14	(1)	90	(16)
C	859	(41)	267	(44)	640	(59)	247	(45)
D	693	(51)	ND	ND	435	(40)	217	(39)

*BMI* body mass index, *ND* no data

^a^Represented as mean ± standard deviation

^b^BMI classification for adults was reported according to WHO [23]

Table 2 shows the mean (SEM), median (50%) and percentile TFI (mL/day) for each country according to gender. The TFI ranged from 1748 mL/day in Mexican women to 2318 mL/day in Argentinean women. There was no significant gender difference in TFI in Mexico, Argentina and Uruguay; however, women drank significantly less than men in Brazil. Between-country comparisons of TFI for each population are shown in Table 2. The highest median (P25–P75) daily TFI was in Argentina, 2133 (1524–2865) mL/day, with Mexico having the lowest intake, 1496 (1069–2150) mL/day. There were significant differences in median TFI between the countries. Both Uruguay and Argentina consumed significantly more fluid per day than Mexico and Brazil, TFI was also significantly higher in Argentina than in Uruguay. The distribution of these data for each country is given in 250 mL/day (an average serving) intervals in the supplementary data (Fig. S1).

**Table 2:** Daily total fluid intake (mL/day) for adults (≥ 18 years) by country and gender

Country	Gender	*N* (%)	Mean ± SEM	Percentiles						
				5	10	25	50	75	90	95
Mexico	Men	746 (44)	1762 ± 35	649	771	1083	1543	2172	3003	3668
	Women	931 (56)	1748 ± 33	624	743	1049	1469	2145	3129	3906
Brazil	Men	224 (47)	1968 ± 71	698	867	1187	1701^a^	2536	3390	4453
	Women	253 (53)	1693 ± 57	589	718	1053	1479	2228	2846	3250
Argentina	Men	464 (43)	2210 ± 47	772	985	1466	2092	2838	3532	4111
	Women	625 (57)	2318 ± 42	885	1097	1563	2174	2884	3725	4392
Uruguay	Men	278 (50)	1979 ± 59	622	805	1224	1833	2594	3329	3797
	Women	276 (50)	2018 ± 62	666	812	1177	1884	2593	3309	4272
Mexico		1677	1754 ± 24	628	753	1060	1496^d,e^	2150	3079	3817
Brazil		477	1822 ± 46	625	790	1098	1568^d,e^	2333	3102	3683
Argentina		1089	2272 ± 31	821	1060	1524	2133^b,c,e^	2865	3630	4270
Uruguay		554	1999 ± 43	638	811	1194	1873^b,c,d^	2593	3311	3916

*SEM* standard error of the mean

^a^Wilcoxon test was used for gender comparisons, and Wilcoxon signed-rank test (*p* < 0.05) was used for between country comparisons

^b^Significantly different from Mexico

^c^Significantly different from Brazil

^d^Significantly different from Argentina

^e^Significantly different from Uruguay

**Fig. 2:**
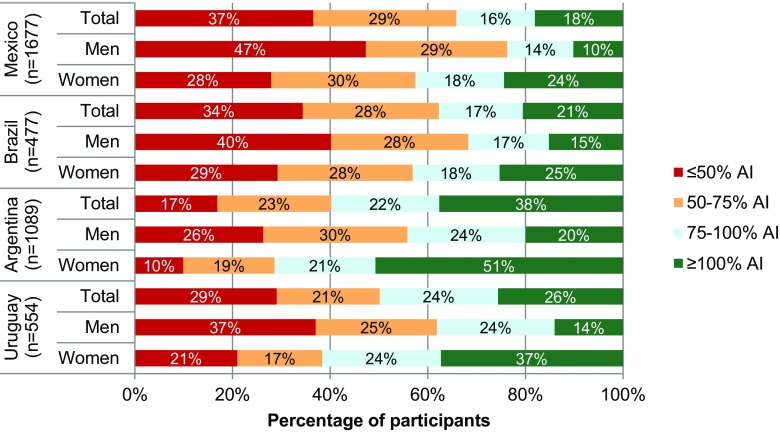
Percentage (%) of adults having adequate intakes (AI) of water from fluids set by the Institute of Medicine [9] based on the 7-day mean of each participant

When compared with the IOM recommendations (adjusted for water from food) [9] (Fig. 2), only 10–25% of the adults surveyed in Mexico and Brazil met the recommendations. This proportion rose to 37% in Uruguayan women and 51% for Argentinian women, while only 14 and 20% of men in these countries, respectively, met the recommended AI for water from fluids. In all four countries, women were more likely than men to meet the recommendations. The percentage of adults drinking ≤ 50% of the recommended intake is particularly high, with nearly half of Mexican adult males (47%) failing to meet 50% of AI. A similar picture was seen in Uruguay and Brazil at 37 and 40%, compared with Argentina where 26% of the male participants had a mean TFI lower than 50% of the recommendation.

**Fig. 3:**
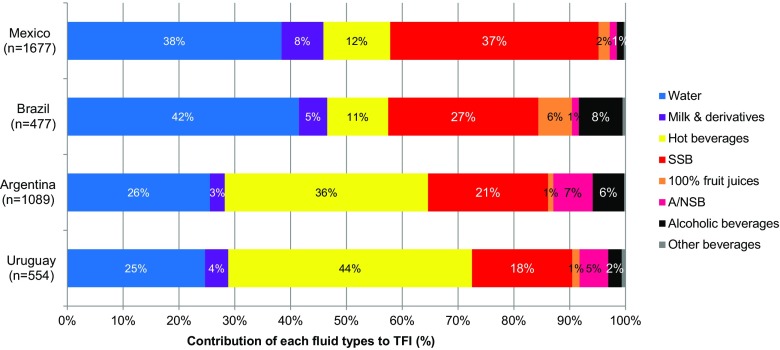
Contribution of different fluid types (%) to total fluid intake among adults (≥ 18 years), by country

**Fig. 4:**
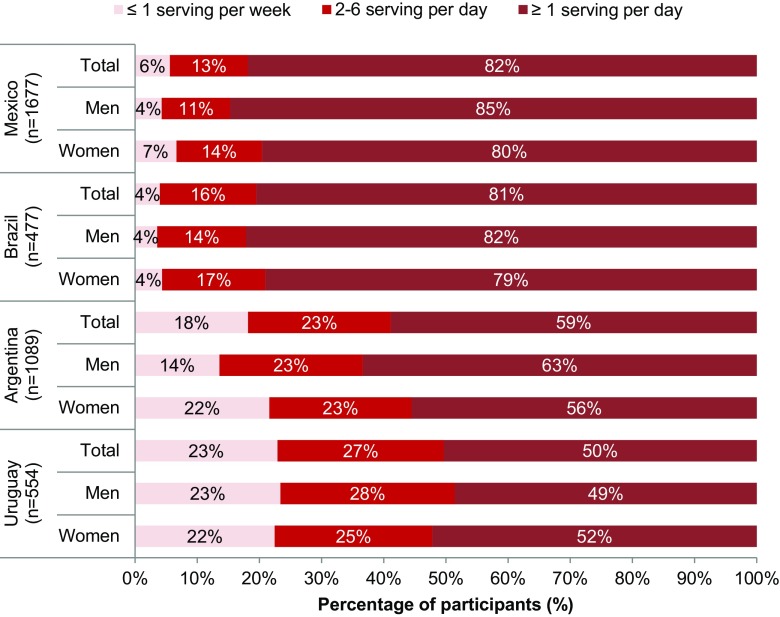
Percentage (%) of adults drinking SSB daily, or less frequently, by country

When the median daily TFI was analyzed by type of beverage consumed (Table 3; Fig. 3), statistically significant differences (*p* < 0.05) were seen between the individual countries. Total fluid intakes in Mexico and Brazil were similar; Argentina and Uruguay were significantly different from each other and the other countries. Although Argentina and Uruguay had the highest median TFI in mL/day, the amount of plain water drunk, either bottled or tap, was less than 400 mL/day, which represents approximately 25% of TFI. Conversely, the majority of daily TFI (≈ 700 mL) was hot beverages (36 and 44% of mean daily TFI, respectively), particularly maté (26 and 37% of mean daily TFI, respectively) unlike in Mexico and Brazil, where less than 150 mL of median TFI was attributable to hot beverages (12% of the mean daily TFI) (Fig. 3). The median TFI and fluid types for each country, according to gender, are shown in supplementary Tables S4a–d. The total volume of SSB consumed in the four countries was significantly different from each other (*p* < 0.05), with Mexico consuming the highest volume [531 (300–895) mL/day], approximately a third of daily TFI (Fig. 3). This was over twice the amount of SSB drunk daily in Uruguay [251 (30–559) mL/day]. In Brazil, SSB consumption was 409 (195–669) mL/day. In addition, over 80% of Brazilian and Mexican adults drank one or more servings per day. This was significantly different from Argentina and Uruguay where 59 and 50% of adults, respectively (*p* < 0.05), drank one or more servings of SSB per day. A consumption of ≤ 1 serving of SSB per week was reported by 6% of Mexican, 4% of Brazilian, 18% of Argentinean and 23% of Uruguayan adults (Fig. 4).

**Table 3:** Median (P25–P75) daily intake (mL/day) of different fluid types and the percentage of consumers among adults (≥ 18 years), by country

	Mexico (*n* = 1677)		Brazil (*n* = 477)		Argentina (*n* = 1089)		Uruguay (*n* = 554)	
	Median (P25–P75)	% Consumers	Median (P25–P75)	% Consumers	Median (P25–P75)	% Consumers	Median (P25–P75)	% Consumers
Water	450 (180–900)^b,c,d^	90	599 (349–1004)^a,c,d^	100	386 (96–855)^a,b^	84	325 (129–721)^a,b^	89
Bottled water	390 (120–831)	86	11 (0–311)	52	0 (0–240)	45	214 (0–596)	68
Tap water	0 (0–0)	18	357 (92–736)	84	129 (0-493)	63	0 (0–109)	36
Milk and derivatives	60 (0–186)^b,c,d^	62	34 (0–117)^a,c,d^	57	0 (0–46)^a,b,d^	32	0 (0–125)^a,b,c^	38
Hot beverages	121 (0–286)^b,c,d^	74	150 (51–263)^a,c,d^	88	732 (431–1136)^a,b^	98	699 (184–1356)^a,b^	86
Coffee	81 (0–250)	68	131 (35–250)	84	71 (0–241)	63	0 (0–129)	41
Tea	0 (0–9)	25	0 (0–0)	23	0 (0–94)	40	0 (0–0)	21
Mate	ND	ND	ND	ND	488 (146–900)	82	514 (0–1204)	63
Other hot beverages	ND	ND	ND	ND	ND	ND	0 (0–0)	1
SSB	531 (300–895)^b,c,d^	94	409 (195–669)^a,c,d^	95	338 (82–754)^a,b,d^	82	215 (30–559)^a,b,c^	77
CSD	171 (0–390)	75	168 (36–353)	80	107 (0–366)	65	50 (0–343)	55
Juice-based drinks	17 (0–161)	52	118 (28–275)	79	0 (0–137)	40	0 (0–20)	26
Functional beverages	0 (0–0)	13	0 (0–0)	13	0 (0–0)	8	0 (0–0)	7
RTD tea and coffee	0 (0–0)	19	0 (0–0)	20	0 (0–0)	2	0 (0–0)	1
Flavored water	99 (0–300)	66	0 (0–0)	18	0 (0–50)	29	0 (0–0)	20
100% fruit juices	0 (0–0)^b,c,d^	23	50 (0–165)^a,c,d^	65	0 (0–0)^a,b^	15	0 (0–0)^a,b^	13
A/NSB	0 (0–0)^b,c,d^	13	0 (0–0)^a,c,d^	22	0 (0–154)^a,b,d^	41	0 (0–110)^a,b,c^	33
Alcoholic beverages	0 (0–0)^b,c,d^	13	0 (0–202)^a,d^	47	0 (0–150)^a,d^	47	0 (0–0)^a,b,c^	24
Other beverages	0 (0–0)^b,c^	7	0 (0–0)^a,c^	10	0 (0–0)^a,b,d^	3	0 (0–0)^c^	7

*SSB* sugar-sweetened beverages, *CSD* carbonated sweetened drinks, *RTD* ready to drink, *A*/*NSB* Artificial/non-nutritive sweeteners beverages, *ND* no data

Wilcoxon signed-rank test (*p* < 0.05) to compare countries

^a^Significantly different from Mexico

^b^Significantly different from Brazil

^c^Significantly different from Argentina

^d^ Significantly different from Uruguay

Figure 3 shows the contribution of each beverage type to the daily TFI. Supplementary Fig. S3 shows these data according to gender and country. Mexican adults have median milk and derivatives (8% of mean daily TFI) twice as high as adults in Argentina and Uruguay (3 and 4% of mean daily TFI, respectively). Brazil consumed more of 100% fruit juice than the other three countries, and Uruguay and Mexico recorded very low alcohol consumption. One interesting difference is in the contribution of A/NSB which is much higher in Argentina and Uruguay (7 and 5%, respectively) compared with Brazil and Mexico where these make up only 1% of the mean TFI.

## Discussion

The data collected in these surveys extends and enriches the information reported previously [7, 26] by focusing specifically on countries in the Latin American region and, for the first time, including data from a Uruguayan population. Using the validated *Liq.In*^*7*^ diary [20], the ranking of the three countries in terms of TFI is the same as previously shown [7, 26], with Argentina having the highest mean daily TFI. Approximately, 75% of the study population did not meet the IOM AI for water from fluids [9]; 18% of Mexicans, 21% of Brazilians, 38% of Argentinians and 26% of Uruguayans met the IOM recommendations. In the previous study when the EFSA recommendations were used [7], 43% of Mexicans, 58% of Brazilians and 64% of Argentinians were shown to adhere to the recommendations [8]. Given the higher AIs for adults from IOM, compared with EFSA, is it not surprising that the present study found that fewer participants met the recommendations. The earlier study had used the EFSA recommendations to facilitate comparisons between the 13 different countries included in the study not to overestimate non-adherence. In the present study, almost 40% of adults in Mexico drank less than 50% of the IOM AI, which must be considered a concern and merits further investigation. A high proportion of adults from each of the four countries failed to meet at least 50% of IOM-recommended intakes, particularly men. It is not possible to draw conclusions about the hydration status of the participants, as no hydration biomarkers were included in the study. However, a proportion of participants would probably be considered low drinkers and therefore may be at increased risk of cardiometabolic diseases [4, 5] and renal disease [27].

Analysis of the type of fluid that contributed to the mean daily TFI clustered these Latin American countries into pairs; Mexico and Brazil vs. Argentina and Uruguay. Over 80% of adults surveyed in Mexico and Brazil consumed one or more servings of SSB daily, and in Mexico the amount of SSB consumed daily exceeded the amount of water consumed, whereas in Argentina and Uruguay these figures were lower, 59 and 50%, respectively. In all countries, carbonated sweetened drinks (CSDs) were the most frequently consumed SSB. This pattern of high SSB consumption is similar to that found in a previous survey in these populations (Uruguay was not included in the earlier study) [26] and also reflects several studies in Mexico that have drawn attention to the increase in SSB consumption and its potential contribution to chronic disease [16, 28–30].

Mexico is widely considered to have the highest consumption of SSB in the world [28]. This has been linked to the transition from traditional nutrition patterns, as shown by decreased expenditure on fruits and vegetables and a move toward more concentrated carbohydrate sources, particularly SSBs [31]. In a previous study [26], SSB consumption in Mexico was shown to contribute approximately equally in terms of volume to daily TFI as water; in the present study, SSB consumption was higher than water intake. However, in 2014 Mexico introduced a tax aimed at reducing SSB consumption, which is predicted to result in a significant reduction in diabetes and cardiovascular disease [32].

Interesting differences were seen between the present data for Brazil and those collected previously [26]. The data presented here show a much greater consumption of SSB, 27% of daily TFI, with 80% of the participants drinking at least one serving per day on average, and a lower consumption of fruit juice. These differences may be explained by the present survey being conducted only in an urban area with participants from a higher overall SES and therefore greater purchasing power and easier access to a greater variety of SSB in terms of proximity to well-stocked local stores in urban areas of Brazil [33].

In Argentina, the contribution of sweet drinks (SSB, A/N SSB and juices) to daily TFI has previously been shown to be greater than that from water [26]; the present survey suggested that slightly more water was consumed proportionally. This was also found to be the case for adults in Uruguay who were not included in the previous study. However, these differences are small and may be due to variations in the study populations; the same validated methodology was used and therefore such differences cannot be methodical in origin. In the present study, Argentina and Uruguay had similar drinking preferences, which were different from Mexico and Brazil in having fewer participants who drank one or more servings of SSB per day and had high maté consumption.

The data from the present study are strengthened by the large numbers of participants and the fact that they were collected using a validated fluid intake diary, the *Liq.In*^7^ record [20], which has been shown to produce accurate and reliable data. The use of harmonized survey methodology and comparable populations, similar in age and gender, has allowed direct and meaningful comparisons between the countries to be made. However, the limitations of the study must be acknowledged. Like all diary assessment methodologies, the *Liq.In*^*7*^ record creates a situation that focuses the participants’ attention on the data being collected and may therefore distort intake. A more valid limitation is the possible bias from having included only one city in Brazil, which may account for some of the differences reported here and the previous *Liq.In*^*7*^ study [26]. In addition, data on food intake were not collected, and therefore information on food moisture was not avaialble. As a result, it is not possible to show whether or not individuals within the lower quartiles of TFI compensated by eating high moisture foods. However, a recent study has shown that low drinkers are unlikely to compensate for low water intakes in this manner [34]. Additionally, hydration markers were not included in the study; therefore, it is not possible to draw conclusions about the hydration status of these populations. In the present study, height and weight were self-reported, as is common in epidemiological studies, and therefore are likely to underestimate overweight and obesity [35]. A considerable proportion (up to 22%) of participants did not report their BMI in Mexico.

## Conclusions

The present study reports data from large surveys conducted on adults in four Latin American countries, namely, Argentina, Brazil, Mexico and Uruguay, using the same methodology. It extends the present knowledge of fluid and types of fluid consumed and compared TFI to recommendations for AI of water from fluids [9]. The majority of adults did not drink as much fluid as is recommended. The intake of SSB remains high, further increasing the risk of obesity [11], type 2 diabetes [12] and cardiovascular disease [13]. While policies are being implemented in some Latin American countries to reduce SSB intake and increase water consumption, data presented here provide further insight into TFI and type of fluid consumed and may aid monitoring of the policy changes. This, and similar studies, will help to guide future public health and health-care policy in these emerging middle-income countries [36].

## Electronic supplementary material

Below is the link to the electronic supplementary material.

Supplementary material 1 (DOCX 96 KB)
