# Supplementary material for: Fluid intake of Latin American adults: results of four 2016 Liq.In7 national cross-sectional surveys
Source: Eur J Nutr. 2018 Jun 1;57(Suppl 3):65–75. doi: 10.1007/s00394-018-1724-z (PMC6008364; doi:10.1007/s00394-018-1724-z)
Supplement: Supplementary file 1 — Supplementary material 1 (DOCX 96 KB) [file 394_2018_1724_MOESM1_ESM.docx]

**FLUID INTAKE OF LATIN AMERICAN ADULTS: RESULTS OF FOUR 2016 LIQ.IN^7^ NATIONAL CROSS-SECTIONAL SURVEYS**

*Martinez H,^1^ Morin C,^2^ Gandy J,^3,4^ Carmuega E,^5^ Arredondo JL,^6^ Pimentel C, ^6^ Moreno LA,^7,8^ Kavouras SA,^9,10^ Salas-Salvadó J, ^8,11^ Guelinckx I,^2*^*

^1^ Hospital Infantil de México Federico Gómez, México City, México

^2^ Department of Hydration & Health, Danone Research, Palaiseau, France^.^

^3^ British Dietetic Association, Birmingham, UK

^4^ School of Life and Medical Sciences, University of Hertfordshire, Hatfield, UK

^5^ Center of Studies on Infant Nutrition (CESNI) Buenos Aires, Argentina

^6^ Unidad de Apoyo a la Investigación Clínica, Instituto Nacional de Pediatría, Mexico City, Mexico

^7^GENUD (Growth, Exercise, NUtrition and Development) Research Group, Faculty of Health Sciences, Universidad de Zaragoza, Instituto Agroalimentario de Aragón (IA2), Instituto Investigación Sanitaria Aragón (IIS Aragón) Zaragoza, Spain

^8^ CIBERobn (Centro de Investigación Biomédica en Red Fisiopatología de la Obesidad y Nutrición), Institute of Health Carlos III, Madrid, Spain

^9^ Hydration Science Lab, University of Arkansas, Fayetteville, AR, USA

^10^ Division of Endocrinology, University of Arkansas for Medical Sciences, Little Rock, AR, USA

^11^ Human Nutrition Unit, Hospital Universitari de Sant Joan de Reus, Faculty of Medicine and Health Sciences, IISPV (Institut d’Investigació Sanitària Pere Virgili), Biochemistry and Biotechnology Department, Universitat Rovira i Virgili, Reus, Spain

*** Corresponding author:** Isabelle GUELINCKX, Hydration and Health department, Danone Research, Route Départemental 128, 91767 Palaiseau, France; [isabelle.guelinckx@danone.com](mailto:clementine.morin@danone.com)

**ONLINE RESOURCES**

**Table S1** Harmonization of the classification of socio-economical status

| Country | Socio-economic status | Socio-economic status for analysis |
| --- | --- | --- |
| **Mexico** | AB | AB |
|  | C+ | C |
|  | C | C |
|  | C- | C |
|  | D+ | D |
|  | D | D |
| **Argentina** | AB | AB |
|  | C1 | C |
|  | C2 | C |
|  | C3 | C |
|  | D1 | D |
|  | D2 | D |
| **Brazil*** | A | AB |
|  | B1 | AB |
|  | B2 | AB |
|  | C1 | C |
|  | C2 | C |
| **Uruguay** | ABC1 | AB |
|  | C2 | C |
|  | C3 | C |
|  | D1 | D |

*Only participants with SES A, B and C were eligible in Sao Paulo (Brazil)

**Table S2** Classification of the fluid types

| Classification of fluids | Detailed Fluid types |
| --- | --- |
| **Water** |  |
| *Bottled water* | Unflavored still water, unflavored sparkling water |
| *Tap water* | Tap water, filtered water, boiled water |
| **Milk & derivatives** | Low fat and full fat milk, raw milk, ready-to-drink flavored milk, homemade flavored milk, yogurt milk, atole/champurrado, powder milk, powder/syrup flavored milk, fruit shake with milk, cocoa compound with milk, vegetal milk (Soya, almond…) |
| **Hot beverages** |  |
| *Coffee* | Coffee, coffee from coffee maker, powder instant coffee, vending machine coffee, restaurant/franchise coffee |
| *Tea* | Homemade hot/cold tea (from tea bags), infusions (herbal), restaurant/franchise tea |
| *Maté* | Mate, mate cocido |
| *Other hot beverages* | Hot Beverages other than coffee, tea or mate. For example *Amargos (Uruguay)* |
| **SSB** |  |
| *Carbonated sweet drinks* (*CSD)* | Cola regular, Concentrated/powder juice with sparkling water, flavored water sparkling, Fruit flavored sparkling |
| *Juice-based drinks* | Eskimo/smoothies (ready to drink or homemade), packaged fruit juice/nectar/vegetable, Fruit shake with water, powder/syrups water, still lemonade |
| *Functional beverages* | Sports Drinks, tonic regular, energy drinks, flavored water enriched with vitamins/minerals, quina water |
| *RTD Tea & Coffee* | Bottled coffee, Ice coffee, ready to drink bottled tea, ready to drink/homemade ice tea, powder tea |
| *Flavored water* | Flavored packaged/homemade water, *aguas frescasagua fresca,*  coconut water |
|  |  |
| **100% fruit juices** | Natural juice (Vegetable/fruit), Bottled 100% fruit juice & vegetables juice, homemade freshly squeezed juice, ready to drink freshly squeezed juice, freshly squeezed juice "take from outside" |
|  |  |
| **A/NSB** | Cola light/zero, flavored water zero/light, fruit flavored light, light bottled tonic, light bottled ice tea, Bottled juice light |
|  |  |
| **Alcoholic beverages** | Beer, cocktails, pure whiskey/vodka/gin, spirit/liquor/digestive, wine, champagne, cider |
|  |  |
| **Other beverages** | Beverages identified by participants as “other than listed above”, packaged soy drinks, agua de arroz (Mexico), diet drinks as meal replacement (slim fast), ready to drink soy based juice, Beer 0% Alcohol |

*SSB* sugar sweetened beverages, *A/NSB* Artifical/non nutritive sweeteners beverages

**Figure S1** Distribution of daily total fluid intake (mL/day) among adults (≥ 18 years), by country

**Figure S2** Percentage of adults having adequate intakes (AI) of water from fluids set by the European Food Safety (EFSA) [7] based on 7-day mean of each participant by gender.

*M* men, *W* women

**Table S3a** Mean (±SEM) daily intake of different fluid types (mL/day) in adults by gender

|  | Mexico | | | | | |
| --- | --- | --- | --- | --- | --- | --- |
|  | Total  (n=1677) | | Men  (n=746) | | Women(n=931) | |
| **TFI** | **1754** | **±24** | **1762** | **±35** | **1748** | **±33** |
| Water | 673 | ±18 | 618 | ±24 | 717 | ±26 |
| *Bottled water* | 611 | ±18 | 577 | ±25 | 639 | ±25 |
| *Tap water* | 62 | ±6 | 42 | ±6 | 78 | ±11 |
| Milk & derivatives | 132 | ±5 | 127 | ±7 | 135 | ±7 |
| Hot beverages | 210 | ±7 | 214 | ±10 | 207 | ±9 |
| *Coffee* | 171 | ±6 | 188 | ±9 | 158 | ±7 |
| *Tea* | 39 | ±3 | 26 | ±3 | 49 | ±5 |
| *Mate* | ND |  | ND |  | ND |  |
| *Other hot beverages* | ND |  | ND |  | ND |  |
| SSB | 655 | ±13 | 704 | ±19 | 615 | ±17 |
| *CSD* | 276 | ±9 | 323 | ±15 | 239 | ±10 |
| *Juice-based drinks* | 118 | ±5 | 126 | ±8 | 111 | ±6 |
| *Functional beverages* | 19 | ±2 | 24 | ±4 | 16 | ±2 |
| *RTD tea & coffee* | 27 | ±2 | 26 | ±4 | 27 | ±3 |
| *Flavored water* | 215 | ±8 | 205 | ±11 | 222 | ±11 |
| 100% fruit juices | 35 | ±3 | 37 | ±4 | 33 | ±3 |
| A/NSD | 23 | ±2 | 24 | ±4 | 22 | ±3 |
| Alcoholic beverages | 22 | ±2 | 33 | ±4 | 13 | ±2 |
| Other beverages | 6 | ±1 | 4 | ±1 | 7 | ±1 |

*TFI* total fluid intake*, SSB* sugar sweetened beverages, *CSD* carbonated sweetened drinks, *RTD* ready to drink, *A/NSB a*rtificial/non-nutritive sweetened beverages, *ND* no data

**Table S3b** Mean (±SEM) daily intake of different fluid types (mL/day) in adults by gender

|  | Brazil | | | | | |
| --- | --- | --- | --- | --- | --- | --- |
|  | Total  (n=477) | | Men  (n=224) | | Women  (n=253) | |
| **TFI** | **1822** | **±46** | **1968** | **±71** | **1693** | **±57** |
| Water | 756 | ±28 | 797 | ±46 | 720 | ±33 |
| *Bottled water* | 239 | ±20 | 230 | ±28 | 247 | ±29 |
| *Tap water* | 517 | ±27 | 567 | ±45 | 473 | ±31 |
| Milk & derivatives | 92 | ±7 | 87 | ±10 | 97 | ±9 |
| Hot beverages | 199 | ±10 | 208 | ±17 | 191 | ±12 |
| *Coffee* | 185 | ±10 | 201 | ±16 | 171 | ±11 |
| *Tea* | 14 | ±2 | 8 | ±2 | 20 | ±3 |
| *Mate* | ND |  | ND |  | ND |  |
| *Other hot beverages* | ND |  | ND |  | ND |  |
| SSB | 490 | ±19 | 532 | ±30 | 453 | ±25 |
| *CSD* | 250 | ±13 | 286 | ±22 | 217 | ±15 |
| *Juice-based drinks* | 192 | ±11 | 204 | ±16 | 182 | ±14 |
| *Functional beverages* | 14 | ±3 | 17 | ±5 | 11 | ±3 |
| *RTD tea & coffee* | 18 | ±3 | 11 | ±2 | 25 | ±5 |
| *Flavored water* | 16 | ±3 | 13 | ±4 | 19 | ±5 |
| 100% fruit juices | 110 | ±7 | 119 | ±11 | 101 | ±9 |
| A/NSB | 23 | ±4 | 19 | ±5 | 26 | ±5 |
| Alcoholic beverages | 142 | ±12 | 193 | ±22 | 98 | ±12 |
| Other beverages | 10 | ±2 | 13 | ±5 | 7 | ±2 |

*TFI* total fluid intake, *SSB* sugar sweetened beverages, *CSD* carbonated sweetened drinks, *RTD* ready to drink, *A/NSB a*rtificial/non-nutritive sweetened beverages, *ND* no data

**Table S3c** Mean (±SEM) daily intake of different fluid types (mL/day) in adults by gender

|  | Argentina | | | | | |
| --- | --- | --- | --- | --- | --- | --- |
|  | Total  (n=1089) | | Men  (n=464) | | Women  (n=625) | |
| **TFI** | **2272** | **±31** | **2210** | **±47** | **2318** | **±42** |
| Water | 581 | ±20 | 571 | ±30 | 588 | ±26 |
| *Bottled water* | 240 | ±15 | 246 | ±24 | 236 | ±19 |
| *Tap water* | 341 | ±16 | 325 | ±23 | 352 | ±21 |
| Milk & derivatives | 60 | ±4 | 73 | ±7 | 51 | ±6 |
| Hot beverages | 827 | ±17 | 676 | ±21 | 939 | ±25 |
| *Coffee* | 155 | ±7 | 163 | ±10 | 149 | ±9 |
| *Tea* | 77 | ±5 | 58 | ±6 | 91 | ±7 |
| *Mate* | 595 | ±17 | 455 | ±21 | 699 | ±25 |
| *Other hot beverages* | ND |  | ND |  | ND |  |
| SSB | 488 | ±16 | 570 | ±27 | 428 | ±19 |
| *CSD* | 259 | ±11 | 333 | ±20 | 203 | ±12 |
| *Juice-based drinks* | 143 | ±10 | 144 | ±15 | 143 | ±13 |
| *Functional beverages* | 12 | ±2 | 14 | ±3 | 10 | ±2 |
| *RTD tea & coffee* | 3 | ±1 | 4 | ±2 | 2 | ±1 |
| *Flavored water* | 72 | ±6 | 75 | ±10 | 70 | ±7 |
| 100% fruit juices | 22 | ±3 | 18 | ±4 | 24 | ±4 |
| A/NSB | 160 | ±10 | 120 | ±13 | 190 | ±14 |
| Alcoholic beverages | 128 | ±8 | 177 | ±14 | 91 | ±8 |
| Other beverages | 6 | ±1 | 5 | ±2 | 7 | ±2 |

*TFI* total fluid intake, *SSB* sugar sweetened beverages, *CSD* carbonated sweetened drinks, *RTD* ready to drink, *A/NSB a*rtificial/non-nutritive sweetened beverages, *ND* no data

**Table S3d** Mean (±SEM) daily intake of different fluid types (mL/day) in adults by gender

|  | Uruguay | | | | | |
| --- | --- | --- | --- | --- | --- | --- |
|  | Total  (n=554) | | Men  (n=278) | | Women  (n=276) | |
| **TFI** | **1999** | **±43** | **1979** | **±59** | **2018** | **±62** |
| Water | 493 | ±22 | 488 | ±31 | 498 | ±32 |
| *Bottled water* | 375 | ±21 | 373 | ±28 | 377 | ±32 |
| *Tap water* | 118 | ±11 | 115 | ±17 | 121 | ±14 |
| Milk & derivatives | 83 | ±6 | 75 | ±8 | 91 | ±10 |
| Hot beverages | 873 | ±35 | 870 | ±49 | 875 | ±49 |
| *Coffee* | 93 | ±7 | 85 | ±10 | 101 | ±11 |
| *Tea* | 44 | ±6 | 31 | ±6 | 57 | ±10 |
| *Mate* | 735 | ±35 | 754 | ±50 | 715 | ±49 |
| *Other hot beverages* | 1 | ±1 | 1 | ±1 | 2 | ±1 |
| SSB | 359 | ±17 | 349 | ±24 | 369 | ±25 |
| *CSD* | 219 | ±14 | 220 | ±19 | 218 | ±21 |
| *Juice-based drinks* | 75 | ±8 | 72 | ±11 | 79 | ±12 |
| *Functional beverages* | 14 | ±3 | 14 | ±4 | 14 | ±4 |
| *RTD tea & coffee* | 1 | ±0 | 1 | ±1 | 1 | ±1 |
| *Flavored water* | 50 | ±7 | 43 | ±9 | 57 | ±10 |
| 100% fruit juices | 26 | ±4 | 27 | ±6 | 26 | ±5 |
| A/NSB | 103 | ±10 | 102 | ±14 | 104 | ±13 |
| Alcoholic beverages | 48 | ±6 | 62 | ±9 | 35 | ±7 |
| Other beverages | 14 | ±3 | 7 | ±2 | 21 | ±7 |

*TFI* total fluid intake, *SSB* sugar sweetened beverages, *CSD* carbonated sweetened drinks, *RTD* ready to drink, *A/NSB a*rtificial/non-nutritive sweetened beverages, *ND* no data

**Table S4a** Median (P25-P75) daily intake (mL/day) of different fluid types and the percentage of consumers in adults by gender

|  | **Mexico (n=1677)** | | | |
| --- | --- | --- | --- | --- |
|  | Men (n=746) | | Women (n=931) | |
|  | **P50 (P25-P75)** | **% consumers** | **P50 (P25-P75)** | **% consumers** |
| Water | 429 (163-846) | 88 | 469 (196-977) | 92 |
| *Bottled water* | *383 (103-800)* | *85* | *404 (132-864)* | *86* |
| *Tap water* | *0 (0-0)* | *17* | *0 (0-0)* | *19* |
| Milk & derivatives | 50 (0-180) | 61 | 64 (0-193) | 63 |
| Hot beverages | 136 (0-315) | 73 | 113 (4-279) | 75 |
| *Coffee* | *101 (0-269)* | *69* | *71 (0-229)* | *66* |
| *Tea* | *0 (0-0)* | *19* | *0 (0-34)* | *31* |
| *Mate* | *ND* | *ND* | *ND* | *ND* |
| *Other hot beverages* | *ND* | *ND* | *ND* | *ND* |
| SSB | 578 (354-951) | 96 | 489 (265-846) | 93 |
| *CSD* | *206 (46-429)* | *79* | *135 (0-343)* | *71* |
| *Juice-based drinks* | *13 (0-175)* | *50* | *21 (0-158)* | *52* |
| *Functional beverages* | *0 (0-0)* | *13* | *0 (0-0)* | *12* |
| *RTD tea & coffee* | *0 (0-0)* | *17* | *0 (0-0)* | *21* |
| *Flavored water* | *85 (0-297)* | *63* | *111 (0-300)* | *68* |
| 100% fruit juices | 0 (0-0) | 22 | 0 (0-0) | 23 |
| A/NSB | 0 (0-0) | 13 | 0 (0-0) | 14 |
| Alcoholic beverages | 0 (0-0) | 15 | 0 (0-0) | 11 |
| Other beverages | 0 (0-0) | 5 | 0 (0-0) | 8 |

*SSB* sugar sweetened beverages, *CSD* carbonated sweetened drinks, *RTD* ready to drink, *A/NSB* artificial/non-nutritive sweetened beverages, *ND* no data

**Table S4b** Median (P25-P75) daily intake (mL/day) of different fluid types and the percentage of consumers in adults by gender

|  | **Brazil (n=477)** | | | |
| --- | --- | --- | --- | --- |
|  | Men (n=224) | | Women (n=253) | |
|  | **P50 (P25-P75)** | **% consumers** | **P50 (P25-P75)** | **% consumers** |
| Water | 629 (345-1034) | 100 | 581 (351-987) | 100 |
| *Bottled water* | *16 (0-312)* | *53* | *7 (0-299)* | *50* |
| *Tap water* | *378 (99-793)* | *85* | *343 (73-688)* | *82* |
| Milk & derivatives | 27 (0-96) | 52 | 36 (0-136) | 62 |
| Hot beverages | 142 (47-272) | 86 | 156 (58-252) | 89 |
| *Coffee* | *130 (36-265)* | *85* | *131 (31-241)* | *84* |
| *Tea* | *0 (0-0)* | *14* | *0 (0-19)* | *30* |
| *Mate* | *ND* | *ND* | *ND* | *ND* |
| *Other hot beverages* | *ND* | *ND* | *ND* | *ND* |
| SSB | 446 (213-696) | 95 | 382 (178-627) | 95 |
| *CSD* | *199 (43-408)* | *82* | *150 (30-304)* | *79* |
| *Juice-based drinks* | *129 (27-296)* | *78* | *107 (29-254)* | *80* |
| *Functional beverages* | *0 (0-0)* | *13* | *0 (0-0)* | *13* |
| *RTD tea & coffee* | *0 (0-0)* | *15* | *0 (0-3)* | *25* |
| *Flavored water* | *0 (0-0)* | *15* | *0 (0-0)* | *20* |
| 100% fruit juices | 50 (0-202) | 63 | 48 (0-147) | 66 |
| A/NSD | *0 (0-0)* | 19 | 0 (0-3) | 25 |
| Alcoholic beverages | 50 (0-254) | 54 | 0 (0-129) | 41 |
| Other beverages | *0 (0-0)* | 10 | *0 (0-0)* | 11 |

*SSB* sugar sweetened beverages, *CSD* carbonated sweetened drinks, *RTD* ready to drink, *A/NSB a*rtificial/non-nutritive sweetened beverages, *ND* no data

**Table S4c** Median (P25-P75) daily intake (mL/day) of different fluid types and the percentage of consumers in adults by gender

|  | **Argentina (n=1089)** | | | |  | |
| --- | --- | --- | --- | --- | --- | --- |
|  | Men (n=464) | | Women (n=625) | | |  |
|  | **P50 (P25-P75)** | **% consumers** | **P50 (P25-P75)** | **% consumers** | |  |
| Water | 372 (64-851) | 80 | 392 (122-864) | 87 | |  |
| *Bottled water* | *0 (0-263)* | *44* | *0 (0-234)* | *46* | |  |
| *Tap water* | *91 (0-471)* | *60* | *150 (0-514)* | *66* | |  |
| Milk & derivatives | 0 (0-67) | 34 | 0 (0-35) | 30 | |  |
| Hot beverages | 604 (351-920) | 96 | 830 (496-1236) | 99 | |  |
| *Coffee* | *71 (0-250)* | *63* | *71 (0-214)* | *63* | |  |
| *Tea* | *0 (0-46)* | *31* | *0 (0-127)* | *46* | |  |
| *Mate* | *373 (35-696)* | *76* | *576 (244-1059)* | *87* | |  |
| *Other hot beverages* | *ND* | *ND* | *ND* | *ND* | |  |
| SSB | 396 (125-841) | 87 | 293 (43-658) | 79 | |  |
| *CSD* | *173 (0-479)* | *72* | *69 (0-300)* | *60* | |  |
| *Juice-based drinks* | *0 (0-137)* | *38* | *0 (0-137)* | *42* | |  |
| *Functional beverages* | 0 (0-0) | *8* | 0 (0-0) | *7* | |  |
| *RTD tea & coffee* | 0 (0-0) | *2* | 0 (0-0) | *3* | |  |
| *Flavored water* | *0 (0-54)* | *29* | *0 (0-50)* | *29* | |  |
| 100% fruit juices | 0 (0-0) | 12 | 0 (0-0) | 16 | |  |
| A/NSB | 0 (0-58) | 31 | 0 (0-226) | 48 | |  |
| Alcoholic beverages | 50 (0-243) | 54 | 0 (0-105) | 41 | |  |
| Other beverages | 0 (0-0) | 3 | 0 (0-0) | 4 | |  |

*SSB* sugar sweetened beverages, *CSD* carbonated sweetened drinks, *RTD* ready to drink, *A/NSB a*rtificial/non-nutritive sweetened beverages, *ND* no data

**Table S4d** Median (P25-P75) daily intake (mL/day) of different fluid types and the percentage of consumers in adults by gender

|  | **Uruguay (n=554)** | | | |
| --- | --- | --- | --- | --- |
|  | Men (n=278) | | Women (n=276) | |
|  | **P50 (P25-P75)** | **% consumers** | **P50 (P25-P75)** | **% consumers** |
| Water | 304 (134-722) | 90 | 343 (129-722) | 88 |
| *Bottled water* | *245 (0-583)* | *69* | *175 (0-608)* | *66* |
| *Tap water* | *0 (0-88)* | *35* | *0 (0-137)* | *37* |
| Milk & derivatives | 0 (0-108) | 35 | 0 (0-142) | 42 |
| Hot beverages | 602 (150-1426) | 85 | 740 (214-1257) | 87 |
| *Coffee* | *0 (0-118)* | *38* | *0 (0-137)* | *43* |
| *Tea* | *0 (0-0)* | *18* | *0 (0-0)* | *25* |
| *Mate* | *514 (0-1295)* | *63* | *508 (0-1186)* | *62* |
| *Other hot beverages* | *0 (0-0)* | *1* | *0 (0-0)* | *1* |
| SSB | 203 (24-532) | 76 | 221 (31-571) | 78 |
| *CSD* | *81 (0-350)* | *56* | *34 (0-300)* | *53* |
| *Juice-based drinks* | *0 (0-0)* | *24* | *0 (0-43)* | *28* |
| *Functional beverages* | *0 (0-0)* | *8* | *0 (0-0)* | *6* |
| *RTD tea & coffee* | *0 (0-0)* | *1* | *0 (0-0)* | *1* |
| *Flavored water* | *0 (0-0)* | *18* | *0 (0-0)* | *22* |
| 100% fruit juices | *0 (0-0)* | 12 | *0 (0-0)* | 14 |
| A/NSB | 0 (0-123) | 32 | *0 (0-100)* | 34 |
| Alcoholic beverages | 0 (0-51) | 29 | *0 (0-0)* | 18 |
| Other beverages | *0 (0-0)* | 6 | *0 (0-0)* | 8 |

*SSB* sugar sweetened beverages, *CSD* carbonated sweetened drinks, *RTD* ready to drink, *A/NSB a*rtificial/non-nutritive sweetened beverages, *ND* no data

**Figure S3** Contribution (%) to total fluid intake of the different fluid types in adults (≥18years), by country and gender.

*M* men, *W* women
